# Small Extracellular Vesicles Released From Human Chemically Induced Liver Progenitors Have the Potential to Improve Liver Fibrosis in Mice

**DOI:** 10.1016/j.gastha.2026.100910

**Published:** 2026-03-03

**Authors:** Tomoko Yamaguchi, Juntaro Matsuzaki, Takeshi Katsuda, Kiminori Kimura, The hCLiP-EV Project Group, Hitoshi Tsugawa, Tohru Kiyono, Masaki Kimura, Marta Prieto-Vila, Yuto Oshima, Yoshimasa Saito, Takahiro Ochiya

**Affiliations:** 1 Division of Pharmacotherapeutics, Keio University Faculty of Pharmacy, Tokyo, Japan; 2 Division of Interdisciplinary Genetics and Nanomedicine, Research Center for Drug Discovery, Keio University Faculty of Pharmacy, Tokyo, Japan; 3 Department of Molecular and Cellular Medicine, Tokyo Medical University, Tokyo, Japan; 4 Department of Chemical System Engineering, Graduate School of Engineering, The University of Tokyo, Tokyo, Japan; 5 Department of Hepatology, Tokyo Metropolitan Cancer and Infectious Diseases Center, Komagome Hospital, Tokyo, Japan

Liver fibrosis requires effective drug treatments because it is a leading cause of liver disease progression and cirrhosis.^1^ Cell therapy research, including mesenchymal stem cells (MSCs), is progressing; however, this approach carries the risk of immune rejection. We successfully generated rodent mature hepatocyte-derived liver progenitor cells, named chemically induced liver progenitors (CLiPs), by reprogramming rodent hepatocytes using small-molecule compounds.^2^ Rat CLiPs ameliorated fibrosis in an animal model that recapitulates metabolic dysfunction–associated steatohepatitis.^3^ We also generated human CLiPs (hCLiPs) by performing small-molecule reprogramming in primary human hepatocytes and diseased livers.^4^^,^^5^ Furthermore, they have a high replacement rate in a transgenic mouse model of chronic liver injury and secrete human albumin. These findings suggest that hCLiPs may display therapeutic potency for chronic liver injury. Many recent reports indicate that MSCs and their small extracellular vesicles (sEVs) act as communication tools among cells. Those have hepatoprotective effects and can overcome the obstacles associated with cell transplantation for clinical applications.^6^^,^^7^ Nonetheless, some reports demonstrate that sEVs derived from tissue-specific stem cells have much stronger antifibrotic effects than those derived from MSCs.^8^^,^^9^ Therefore, we investigated the therapeutic potential of hCLiPs and hCLiP-derived sEVs (hCLiP-sEVs) in liver fibrosis and aimed to elucidate the mechanism of action.

hCLiPs were intrasplenically administered to mice with CCl_4_-induced liver fibrosis, and regression was evaluated (Supplementary Figure 1A). A decreased hepatic hydroxyproline content and pathological improvement by immunohistochemistry of collagen type 1A were observed in the hCLiP transplantation group (Figure 1A and B). hCLiP transplantation decreased mRNA expression of pro-fibrogenic markers such as *Acta2*, *Col1a1*, and *Timp1*, while it increased mRNA expression of antifibrotic markers such as *Mmp2* (Figure 1C). We attempted to detect human cells in the mouse liver, and by digital polymerase chain reaction (PCR), 0% to 1% human cells were detected 2 weeks after transplantation (Supplementary Figure 1B and C). These results indicate that hCLiP transplantation ameliorates liver fibrosis, and we hypothesized this may be due to the antifibrotic effect of sEVs secreted by hCLiPs, which partially resided in the liver.

**Figure 1:**
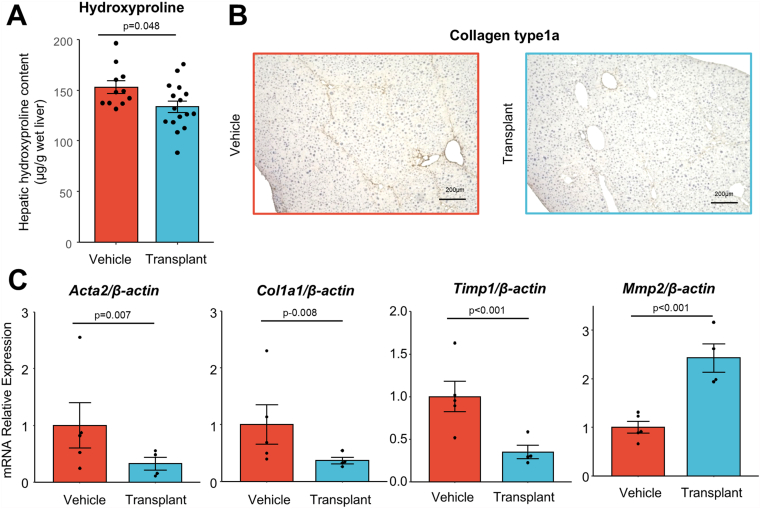
hCLiP transplantation improves liver fibrosis in mice. (A) The hydroxyproline content of the mouse liver was decreased in the transplantation group. (B) Immunostaining showed that transplantation reduced collagen type 1A expression. (C) mRNA expression analysis showed that transplantation upregulated *Mmp2*, an antifibrotic gene, while downregulating profibrotic genes, *Acta2*, *Col1a1*, and *Timp1*.

hCLiP can proliferate, but nonhepatic cells with a fibroblast-like morphology were also observed, and the percentage of these cells changed among repeated passages, as assessed by flow cytometric analysis of the epithelial cell surface marker proteins EPCAM and CD24.^5^ Thus, maintaining hCLiPs with optimal functionality over the long term remained challenging. To consistently collect hCLiP-sEVs of uniform quality, hCLiPs were immortalized by overexpressing the *CDK4*^*R24C*^, *Cyclin D1*, and *TERT* genes (Supplementary Figure 2A). We chose cells with the highest hepatic identities, specifically those that exhibited human albumin expression by single-cell sorting. sEVs were isolated from the culture supernatant of these immortalized hCLiPs by ultracentrifugation. Transmission electron microscopy revealed the characteristic morphology of sEVs, with diameters less than 200 nm within the MISEV2023 definition of small EVs, and this was further validated by nanoparticle tracking analysis (Supplementary Figure 2B and C). The typical EV markers CD9, CD63, and CD81 were detected in the sEV fraction by immunoblotting, while the absence of the Golgi apparatus marker, GM130, was present only in the cell lysate. In addition, proteins encoded by the 3 immortalizing genes were present in the cell lysate, but not in hCLiP-sEVs (Supplementary Figure 2D and E), indicating a low risk of tumorigenesis of these sEVs. The microRNA (miRNA) content of hCLiP-sEVs collected from immortalized hCLiPs did not significantly differ from that of the preimmortalization counterpart (Supplementary Figure 2F).

To evaluate the effects of hCLiP-sEVs on other cells, we conducted in vitro experiments to examine their interactions with hepatic stellate cells (HSCs), the primary contributors to fibrogenesis. Human primary HSCs were activated using transforming growth factor β (TGF-β) (5 ng/mL), and the mRNA expression levels of *ACTA2* and *COL1A1* in HSCs were evaluated by quantitative PCR. Upon hCLiP exposure, the expression of *ACTA2* and *COL1A1* in TGF-β-activated HSCs significantly decreased (Figure 2A and B). To examine if the RNA cargo of hCLiP-sEVs is responsible for the antifibrotic effects of hCLiPs, we transfected TGF-β-activated HSCs with the total RNA extracted from hCLiP-sEVs. The expression levels of *ACTA2* and *COL1A1* in these transfected HSCs were almost the same as those in HSCs exposed to hCLiP-sEVs (Figure 2A and B). Since hCLiP-sEV exposure did not affect HSCs confluency in vitro, the antifibrotic effect is likely mediated by the inactivation of myofibroblasts rather than the induction of apoptosis. Next, we focused on miRNAs, the best-characterized functional RNAs present in sEVs. miRNA sequencing and quantitative PCR showed that miR-122-5p and miR-29a-3p, which are miRNAs that elicit antifibrotic effects, were abundant in hCLiP-sEVs (Supplementary Figure 3A and B). We transfected TGF-β-activated HSCs with a miR-122-5p or miR-29a-3p mimic. Expression of *ACTA2* and *COL1A1* in HSCs significantly decreased upon transfection of the miR-122-5p and miR-29a-3p mimics, respectively (Figure 2C, Supplementary Figure 3C). Finally, to determine whether miR-122-5p and miR-29a-3p in hCLiP-sEVs are responsible for the effects of hCLiPs, we transfected TGF-β-activated HSCs with a miR-122-5p or miR-29a-3p inhibitor and exposed them to hCLiP-sEVs. The decreases in expression of *ACTA2* and *COL1A1* in HSCs following hCLiP-sEV exposure were abrogated upon transfection of these miRNA inhibitors, respectively (Figure 2D).

**Figure 2:**
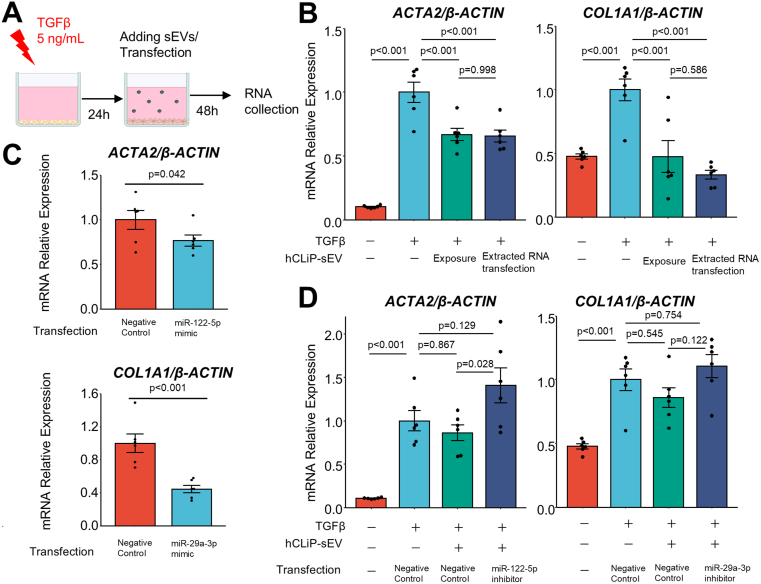
hCLiP-sEVs ameliorate liver fibrosis through their RNA cargo, particularly miR-122-5p and miR-29a-3p. (A) sEVs derived from immortalized hCLiPs suppressed the profibrotic phenotype of human HSCs. Transfection of total RNA extracted from hCLiP-sEVs induced similar levels of *ACTA2* and *COL1A1* expression as hCLiP-sEV exposure. (B) Schematic protocol for hCLiP-sEV exposure and transfection. (C) Transfection of the miR-122-5p and miR-29a-3p mimics to HSCs decreased the expression of *ACTA2* and *COL1A1*, respectively. (D) Transfection of a miR-122-5p and miR-29a-3p inhibitor abrogated the decrease in expression of *ACTA2* and *COL1A1* in HSCs following exposure to hCLiP-sEVs, respectively.

In summary, our results indicate that hCLiPs and hCLiP-sEVs exert an antifibrotic effect through their RNA cargo, particularly miR-122-5p and miR-29a-3p (Supplementary Figure 3D). Cell-free sEV therapy may offer a safer alternative to cell transplantation, with reduced risks of immune rejection and tumorigenicity, although long-term safety profiles remain to be fully established. Therefore, sEVs have been proposed as a novel cell-free therapy for liver fibrosis, and hCLiPs are a promising antifibrotic modality. Both miR-122-5p and miR-29a-3p elicit antifibrotic effects. Furthermore, miR-122-5p is specifically expressed in the liver and is therefore absent in sEVs derived from MSCs.^10^ hCLiP-sEVs are immortalized and can be stably cultured on a large scale in culture medium supplemented with small-molecule compounds, eliminating the need for specialized culture procedures. For these reasons, we propose that hCLiPs provide a more promising sEV source for clinical applications than other cell types. In this report, we revealed that exposure to hCLiP-sEVs suppressed expression of both *ACTA2* and *COL1A1*, which indicates the importance of including a diverse range of miRNAs; however, our focus was limited to 2 well-known miRNAs. Other miRNAs and mRNAs may also contribute to the antifibrotic effect of hCLiPs. While the CCl_4_ model effectively mimics fibrogenesis, we acknowledge that it does not fully recapitulate the metabolic complexity of human metabolic dysfunction–associated steatotic liver disease/metabolic dysfunction–associated steatohepatitis. In the future, it will be essential to further elucidate the mechanism of action by directly comparing miRNA therapies and hCLiP-sEVs and to determine which has the greater therapeutic effect using metabolic fibrosis models.

## Supplementary Materials

Supplementary Materials

Extended PDF
