# Supplementary material for: Small Extracellular Vesicles Released From Human Chemically Induced Liver Progenitors Have the Potential to Improve Liver Fibrosis in Mice
Source: Gastro Hep Adv. 2026 Mar 3;5(5):100910. doi: 10.1016/j.gastha.2026.100910 (PMC13066956; doi:10.1016/j.gastha.2026.100910)
Supplement: Supplementary Materials [file mmc1.pdf]

Supplementary Materials

Cell culture

Primary human hepatocytes (PHHs) (lot FCL) were purchased from Veritas Corporation. The basal medium for culture of PHHs (SHM medium) was prepared following a previous report[1]. Depending on the experiment, this basal medium for hCLiPs was supplemented with 10% KnockOut Serum Replacement (Thermo Scientific) and small molecules, namely, 0.5 μM A-83-01 (Wako) and 3 μM CHIR99021 (Selleck). HHSteC were purchased from ScienCell Research Laboratories and maintained in Stellate Cell Medium containing Stellate Cell Growth Supplement, 2% fetal bovine serum, and penicillin/streptomycin (P/S) (Science Cell Research Laboratories). LX-2 cells were purchased from Sigma and maintained in DMEM, high glucose (Nacalai Tesque, Inc.) containing fetal bovine serum (Biowest) amd P/S (Nacalai Tesque, Inc.).

Induction of hCLiPs from PHHs

hCLiPs were generated from PHHs (lot FCL), which has high hepatic identity in a previous report[1].

hCLiP transplantation into mice with liver fibrosis

To induce liver fibrosis, CCl_4 (0.5 mL/kg) (Wako) dissolved in olive oil (Wako) was administered intraperitoneally twice per week for 8 weeks to 8-week-old NOD-SCID mice. hCLiPs were pelleted

using TrypLE Express (Life Technologies) and suspended in DMEM (Life Technologies). Under isoflurane (Pfizer) anesthesia, the spleen was exposed and 5×10^5 cells were injected into each mouse. Two weeks after transplantation, mice were sacrificed and the degree of liver fibrosis was evaluated. The dosage and schedule were determined based on established protocols for liver cell transplantation[2].

Hydroxyproline quantification

Hydroxyproline in liver tissue was quantified using a Hydroxyproline Assay Kit (BioVision) according to the manufacturer’s instructions.

Digital PCR

Total DNA was extracted from frozen liver tissue using a DNeasy Blood & Tissue Kit (Qiagen). To analyze human cells in mouse liver after hCLiP transplantation, mouse liver following hCLiP transplantation were analyzed using total DNA, the TaqMan Copy Number Reference Assay, Mouse Tfrc (VIC), and probes from a TaqMan RNase P Detection Reagents Kit (FAM). Digital PCR detection was performed using the QuantStudio 3D Digital PCR System (Thermo Fisher Scientific) with PCR Master Mix v2.

Immunohistochemistry

Following dewaxing and rehydration, heat-induced epitope retrieval was performed in ImmunoSaver (Nissin EM) at 98°C for 45 min. Endogenous peroxidase was inactivated with methanol containing 0.3% H_2O_2 at room temperature for 30 min. Thereafter, specimens were permeabilized with 0.1% Triton X-100, treated with Blocking One solution at 4°C for 30 min, and incubated with primary antibodies at 4°C overnight (Supplementary Table). Sections were stained using ImmPRESS IgG-Peroxidase Kits (Vector Labs) and a Metal Enhanced DAB Substrate Kit (Life Technologies) according to the manufacturers' instructions.

Establishment of immortalized hCLiPs

hCLiPs were immortalized by overexpressing the $CDK4^{R24C}$, Cyclin D1, and TERT genes located downstream of the EpCAM promoter using the lentiviral vector plasmids CSII-EpCAMp-tetOff-Adv, CSII-TRE-Tight-cyclin D1, and CSII-TRE-Tight-$CDK4^{R24C}$ [3]. Cell sorting was performed using a FACSAria Ⅲ instrument (BD Biosciences). Cells were labeled with propidium iodide (BD Biosciences) and sorted into 96-well plates.

Albumin enzyme-linked immunosorbent assay

Human albumin in the culture supernatant was quantified using a Human Albumin ELISA

Quantitation Kit (Bethyl) according to the manufacturer's instructions.

Collection of sEVs

hCLiPs were suspended in SHM medium containing 10% KnockOut Serum Replacement, A-83-01, and CHIR99021, and seeded at a density of 3 × 10^4 viable cells/cm^2. The medium was replaced with SHM containing A-83-01 and CHIR99021 (without KnockOut Serum Replacement) on the second day of culture. The culture supernatant was collected after 48 h of culture and centrifuged at 2,000 g for 10 min at 4°C, and the supernatant was filtered using a 0.22 μm filter. The pretreated culture supernatant was ultracentrifuged at 35,000 rpm for 1 h and 10 min at 4℃. Immediately after ultracentrifugation, the supernatant was discarded and sEVs were pelleted (ultracentrifugation was sometimes repeated depending on the amount of culture supernatant). The pellet was resuspended in phosphate-buffered saline (PBS), the sample was ultracentrifuged again, and the supernatant was discarded. The pellet was washed and resuspended in the small amount of PBS left in the tube to create the sEV solution. The collection method, validation, and quantification methods suffice the MISEV2023 guidelines[4].

Nanoparticle tracking analysis

To quantify the particle number in EV samples, nanoparticle tracking analysis was performed

using NanoSight NS300.

Transmission electron microscopy (TEM)

hCLiP-sEVs were fixed by resuspending them in TEM sample buffer containing 1% glutaraldehyde followed by incubation overnight at 4°C. The sample was pipetted onto a Formvar-coated 200-mesh nickel grid (Ted Pella Inc.) and allowed to settle for 25 min. The grid was air-dried and then TEM images were obtained using a JEM-1400 transmission electron microscope (JEOL) at 100 kV.

Protein extraction

Cells were lysed with M-PER Mammalian Protein Extraction Reagent (Thermo Fisher Scientific) by thoroughly pipetting. The lysate was centrifuged at 15,000 g for 10 min at 4°C, and the supernatant was used as the protein solution. The protein concentration was measured using a Pierce BCA Protein Assay Kit (Thermo Fisher Scientific) according to the manufacturer's instructions.

Immunoblotting

The protein solution was mixed with 4× SDS Sample Buffer (Millipore) and incubated at 95°C for

5 min. The sample and molecular weight marker were loaded into 4–20% Mini-PROTEAN TGX Precast Protein Gels (Bio-Rad). Electrophoresis was performed for 45 min at 150 V. Gels were transferred to an Immobilon-P membrane (Merck) for 1 h at 100 V. Blocking was performed and the membrane was incubated with the diluted primary antibody overnight at 4°C (Supplementary Table). After washing, the membrane was incubated with the diluted secondary antibody for 1 h at room temperature. The membrane was washed and stained with ECL Select Western Blotting Detection Reagent (Cytiva). Signals were detected with a Molecular Imager ChemiDoc XRS System (Bio-Rad).

miRNA-sequencing

Libraries were constructed using a QIAseq miRNA Library Kit (Qiagen) according to the manufacturer's protocols. The pooled libraries were sequenced using QIAseq miRNA NGS 96 Index IL (Qiagen) in 75-bp single-end reads. Then, the original FASTQ files generated by CLC Genomics Workbench 22.0.2 were aligned to the miRBase v22 databank.

Exposure of HSCs to hCLiP-sEVs

HHSteCs were cultured overnight and then the medium was replaced with Stellate Cell Medium containing P/S and TGF-β (5 ng/mL) (PeproTech Inc.). After incubation for 24 h, HHSteCs were

exposed to hCLiP-sEVs for 48 h.

Transfection

Transfection of hsa-miR-122-5p inhibitor (MH11012, Thermo Fisher Scientific), hsa-miR-29a-3p inhibitor (MH12499, Thermo Fisher Scientific), hsa-miR-122-5p mimic (MC11012, Thermo Fisher Scientific), and hsa-miR-29a-3p mimic (MC12499, Thermo Fisher Scientific) was performed using jetPRIME (Polyplus) according to the manufacturer's instructions.

qRT-PCR

Total RNA was isolated using an miRNeasy Mini Kit (Qiagen). Reverse transcription was performed using a High-Capacity cDNA Reverse Transcription Kit (Life Technologies) according to the manufacturer's instructions. cDNA was used for PCR with PowerUp SYBR Green Master Mix (Applied Biosystems) using specific primers (Mmp2 forward, ACACTTTCTATGGCTGCCCC; Mmp2 reverse, GTTTCAGGGTCCAGGTCAGG; Timp1 forward, GTAATGCGTCCAGGAAGCCT; Timp1 reverse, GGGGGCCATCATGGTATCTG; Acta2 forward GGCATCATCACCAACTGGGA; Acta2 reverse, AGAGGCATAGAGGGACAGCA; Col1a forward, TTCTCCTGGCAAAGACGGAC; Col1a reverse, CTCAAGGTCACGGTCACGAA; β-actin forward, TCGTGCGTGACATCAAAGAGA; β-actin reverse, GCCACAGGATTCCATACCCAA; β-ACTIN

forward, AGCACTGTGTTGGCGTACAG; β-ACTIN reverse, ACTCTTCCAGCCTTCCTTCC; ACTA2 forward, CTGTTCCAGCCATCCTTCAT; ACTA2 reverse, GGCAATGCCAGGGTACATAG; COL1A1 forward, AACATGACCAAAAACCAAAAGTG; and COL1A1 reverse, CATTGTTTCCTGTGTCTTCTGG).

Statistics

Two groups were compared using the T test. Three groups were compared using a one-way analysis of variance, and, assuming equality of variance was suspected, Tukey's test was performed with the linear mixed models using IBM SPSS Statistics 23 (SPSS Inc.).

Supplementary Figure Legends

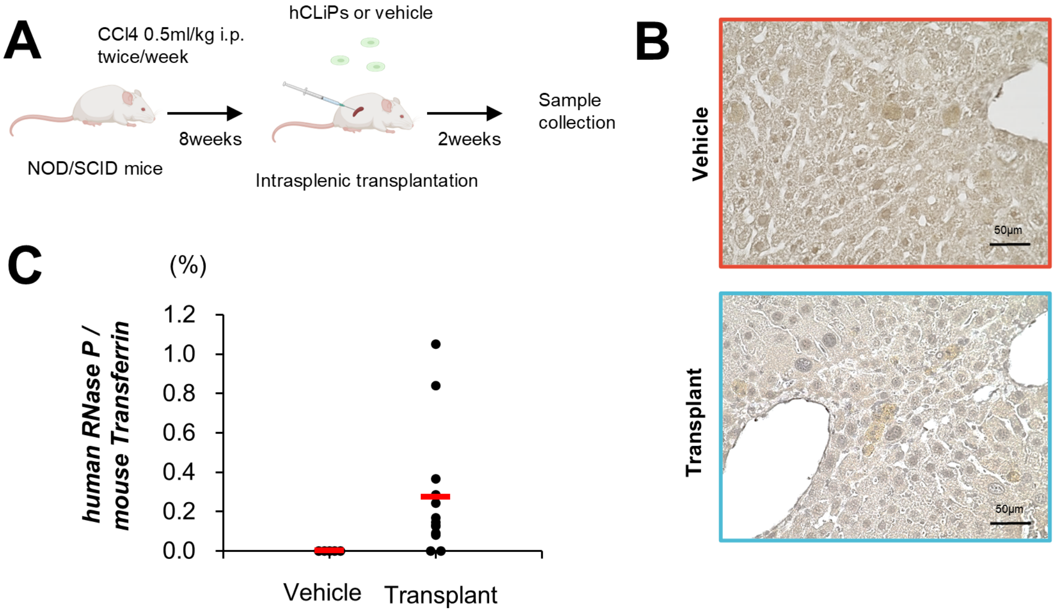

Supplementary Figure 1. The detection of human cells in the mouse tissues confirmed the successful engraftment of the transplanted hCLiP.

(A) Schematic protocol for hCLiP transplantation in a mouse model of liver fibrosis. (B) Immunohistochemistry of human mitochondria. (C) ddPCR analysis of human RNase P/mouse transferrin expression.

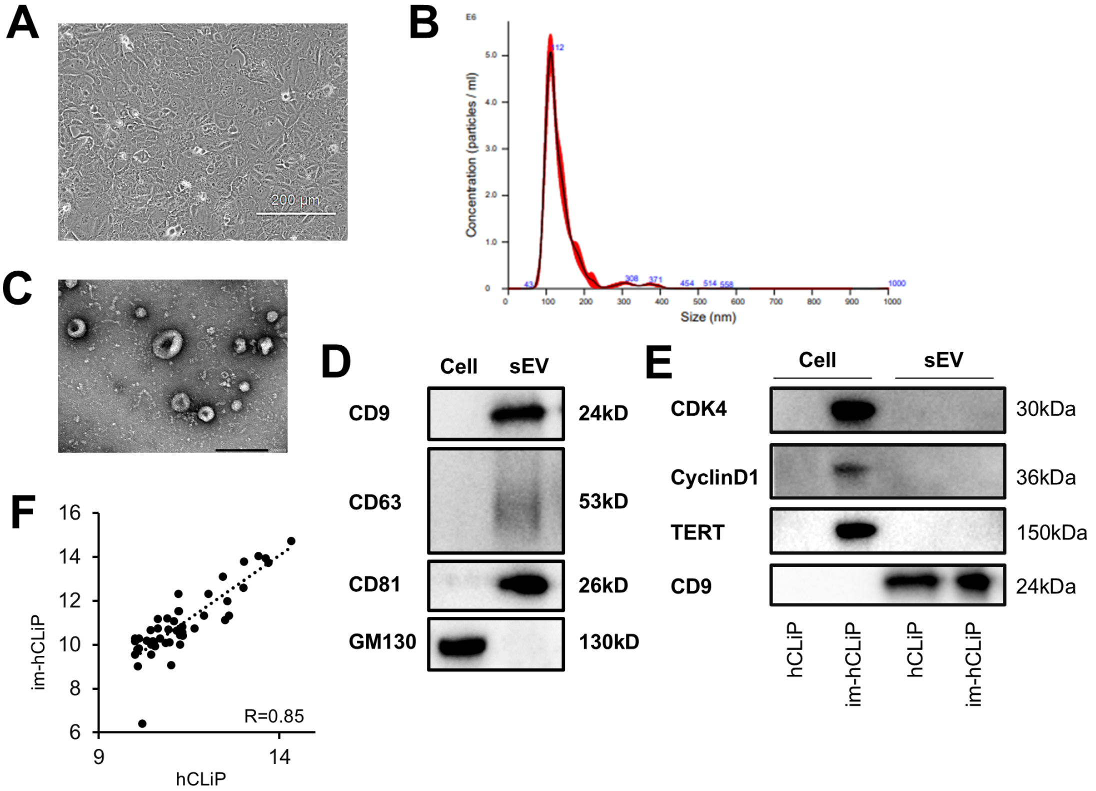

Supplementary Figure 2. Immortalized hCLiP allowed for the proper collection of sEVs, which exhibited characteristics comparable to the original hCLiP.

(A) Cell morphology. (B) Nanoparticle tracking analysis. (C) TEM. (D) Immunoblot of EV marker proteins. (E) Immunoblot of overexpressed genes. (F) miRNA sequencing.

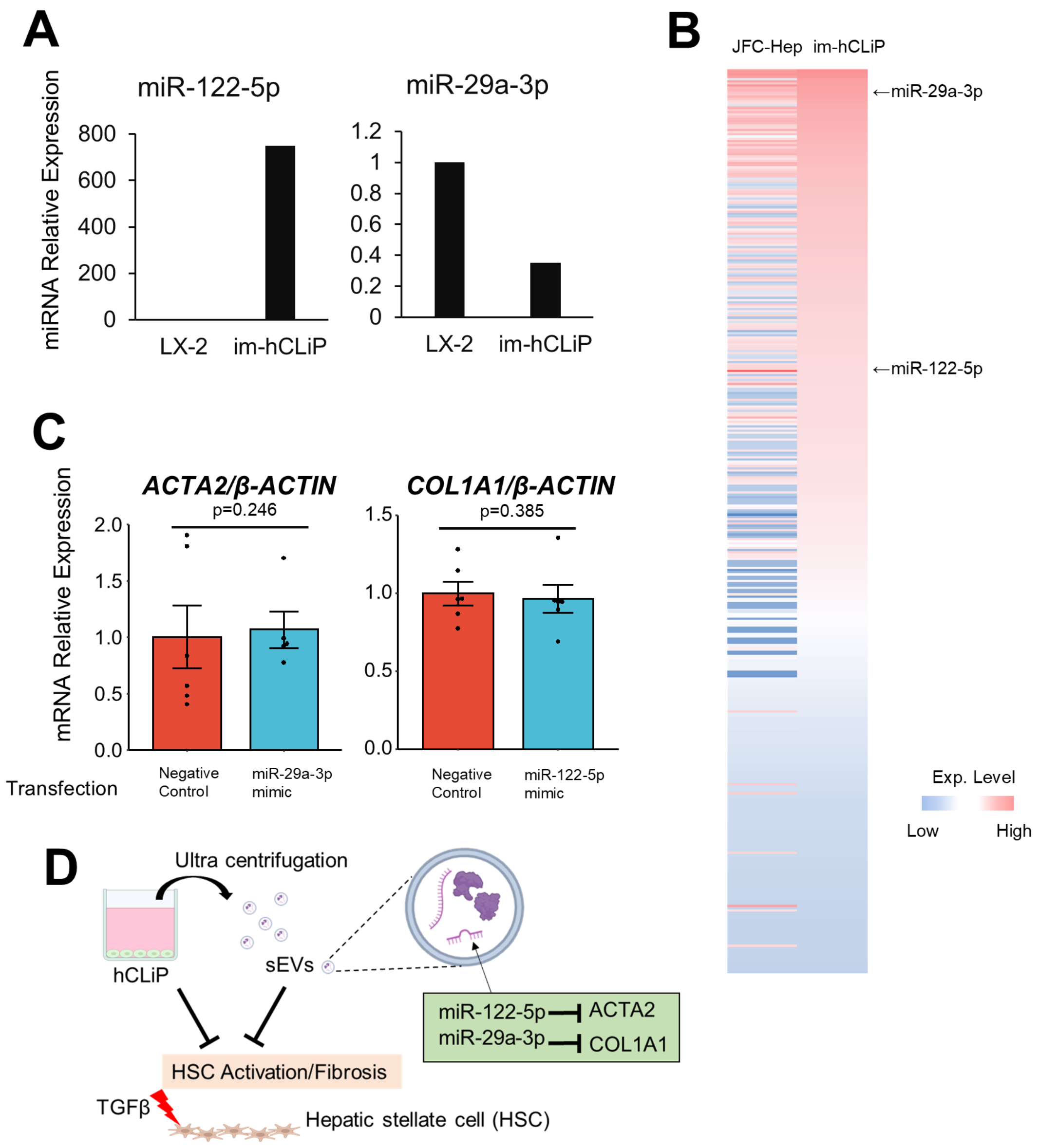

Supplementary Figure 3. The mechanism of action of hCLiP is mainly through EVs, with miR-122-5p and miR-29a-3p playing key roles in this mechanism.

(A) qPCR analysis of miR-122-5p and miR-29a-3p expression. (B) miRNA sequencing. (C) Transfection of a miR-122-5p and miR-29a-3p mimic did not affect expression of *COL1A1* and *ACTA2*, respectively. (D) Schematic overview of the study design.

Supplementary Table. List of antibodies using for immunohistochemistry and immunoblotting.

Antibody	Host animal	Catalog #	Dilution	Dilution buffer	Manufacturer
Col1a	Goat	1310-01	1/200	DAKO real diluent	Southern Biotech
CD9	Mouse	312102	1/5000	Primary: TBS-T containing 10% Blocking One solution<br>Secondary: TBS-T	BD Biosciences
CD63	Mouse	556019	1/2000	Primary: TBS-T containing 10% Blocking One solution<br>Secondary: TBS-T	BD Biosciences
CD81	Mouse	555675	1/5000	Primary: TBS-T containing 10% Blocking One solution<br>Secondary: TBS-T	BioLegend
GM130	Rabbit	11308-1-AP	1/5000	Primary: TBS-T containing 10% Blocking One solution<br>Secondary: TBS-T	Proteintech
CDK4	Rabbit	12790S	1/1000	Primary: TBS-T containing 10% Blocking One solution<br>Secondary: TBS-T	Cell Signaling
Cyclin D1	Mouse	554180	1/1000	Primary: Can Get Signal Solution 1<br>Secondary: Can Get Signal Solution 2	BD Biosciences
TERT	Rabbit	ABE2075	1/1000	Primary: 3% skim milk solution prepared in PBS-T<br>Secondary: 3% skim milk solution prepared in PBS-T	Sigma

References

1. Katsuda T, Matsuzaki J, Yamaguchi T, et al. Generation of human hepatic progenitor cells with regenerative and metabolic capacities from primary hepatocytes. Elife 2019;8:e47313, doi:10.7554/eLife.47313

2. Watanabe Y, Tsuchiya A, Seino S, et al. Mesenchymal Stem Cells and Induced Bone Marrow-Derived Macrophages Synergistically Improve Liver Fibrosis in Mice. Stem Cells Transl Med 2019;8(3):271-284, doi:10.1002/sctm.18-0105

3. Nishiwaki M, Toyoda M, Oishi Y, et al. Immortalization of human hepatocytes from biliary atresia with CDK4. Sci Rep 2020;10(1):17503, doi:10.1038/s41598-020-73992-3

4. Welsh JA, Goberdhan DCI, O'Driscoll L, et al. Minimal information for studies of extracellular vesicles (MISEV2023): From basic to advanced approaches. J Extracell Vesicles 2024;13(2):e12404, doi:10.1002/jev2.12404
