# Supplementary material for: Small Extracellular Vesicles Released From Human Chemically Induced Liver Progenitors Have the Potential to Improve Liver Fibrosis in Mice
Source: Gastro Hep Adv. 2026 Mar 3;5(5):100910. doi: 10.1016/j.gastha.2026.100910 (PMC13066956; doi:10.1016/j.gastha.2026.100910)
Supplement: Extended PDF [file mmc2.pdf]

RESEARCH LETTERS

Small Extracellular Vesicles Released From Human Chemically Induced Liver Progenitors Have the Potential to Improve Liver Fibrosis in Mice

Liver fibrosis requires effective drug treatments because it is a leading cause of liver disease progression and cirrhosis.[1] Cell therapy research, including mesenchymal stem cells (MSCs), is progressing; however, this approach carries the risk of immune rejection. We successfully generated rodent mature hepatocyte-derived liver progenitor cells, named chemically induced liver progenitors (CLiPs), by reprogramming rodent hepatocytes using small-molecule compounds.[2] Rat CLiPs ameliorated fibrosis in an animal model that recapitulates metabolic dysfunction–associated steatohepatitis.[3] We also generated human CLiPs (hCLiPs) by performing small-molecule reprogramming in primary human hepatocytes and diseased livers.[4,5] Furthermore, they have a high replacement rate in a transgenic mouse model of chronic liver injury and secrete human albumin. These findings suggest that hCLiPs may display therapeutic potency for chronic liver injury. Many recent reports indicate that MSCs and their small extracellular vesicles (sEVs) act as communication tools among cells. Those have hepatoprotective effects and can overcome the obstacles associated with cell transplantation for clinical applications.[6,7] Nonetheless, some reports demonstrate that sEVs derived from tissue-specific stem cells have much stronger antifibrotic effects than those derived from MSCs.[8,9] Therefore, we investigated the therapeutic potential of hCLiPs and hCLiP-derived sEVs (hCLiP-sEVs) in liver fibrosis and aimed to elucidate the mechanism of action.

hCLiPs were intrasplenically administered to mice with CCl_4-induced liver fibrosis, and regression was evaluated (Supplementary Figure 1A). A decreased hepatic hydroxyproline content and pathological improvement by immunohistochemistry of collagen type 1A were observed in the hCLiP transplantation group (Figure 1A and B). hCLiP transplantation decreased mRNA expression of pro-fibrogenic markers such as *Acta2*, *Col1a1*, and *Timp1*, while it increased mRNA expression of antifibrotic markers such as *Mmp2* (Figure 1C). We attempted to detect human cells in the mouse liver, and by digital polymerase chain reaction (PCR), 0% to 1% human cells were detected 2 weeks after transplantation (Supplementary Figure 1B and C). These results indicate that hCLiP transplantation ameliorates liver fibrosis, and we hypothesized this may be due to the antifibrotic effect of sEVs secreted by hCLiPs, which partially resided in the liver.

hCLiP can proliferate, but nonhepatic cells with a fibroblast-like morphology were also observed, and the percentage of these cells changed among repeated passages, as assessed by flow cytometric analysis of the epithelial cell surface marker proteins EPCAM and CD24.[5] Thus, maintaining hCLiPs with optimal functionality over the long term remained challenging. To consistently collect hCLiP-sEVs of uniform quality, hCLiPs were immortalized by overexpressing the $CDK4^{R24C}$, *Cyclin D1*, and *TERT* genes (Supplementary Figure 2A). We chose cells with the highest hepatic identities, specifically those that exhibited human albumin expression by single-cell sorting. sEVs were isolated from the culture supernatant of these immortalized hCLiPs by ultracentrifugation. Transmission electron microscopy revealed the characteristic morphology of sEVs, with diameters less than 200 nm within the MISEV2023 definition of small EVs, and this was further validated by nanoparticle tracking analysis (Supplementary Figure 2B and C). The typical EV markers CD9, CD63, and CD81 were detected in the sEV fraction by immunoblotting, while the absence of the Golgi apparatus marker, GM130, was present only in the cell lysate. In addition, proteins encoded by the 3 immortalizing genes were present in the cell lysate, but not in hCLiP-sEVs (Supplementary Figure 2D and E), indicating a low risk of tumorigenesis of these sEVs. The microRNA (miRNA) content of hCLiP-sEVs collected from immortalized hCLiPs did not significantly differ from that of the preimmortalization counterpart (Supplementary Figure 2F).

To evaluate the effects of hCLiP-sEVs on other cells, we conducted in vitro experiments to examine their interactions with hepatic stellate cells (HSCs), the primary contributors to fibrogenesis. Human primary HSCs were activated using transforming growth factor β (TGF-β) (5 ng/mL), and the mRNA expression levels of *ACTA2* and *COL1A1* in HSCs were evaluated by quantitative PCR. Upon hCLiP exposure, the expression of *ACTA2* and *COL1A1* in TGF-β-activated HSCs significantly decreased (Figure 2A and B). To examine if the RNA cargo of hCLiP-sEVs is responsible for the antifibrotic effects of hCLiPs, we transfected TGF-β-activated HSCs with the total RNA extracted from hCLiP-sEVs. The expression levels of *ACTA2* and *COL1A1* in these transfected HSCs were almost the same as those in HSCs exposed to hCLiP-sEVs (Figure 2A and B). Since hCLiP-sEV exposure did not affect HSCs confluency in vitro, the antifibrotic effect is likely mediated by the inactivation of myofibroblasts rather than the induction of apoptosis. Next, we focused on miRNAs, the best-characterized functional RNAs present in sEVs. miRNA sequencing and quantitative PCR showed that miR-122-5p and miR-29a-3p, which are miRNAs that elicit antifibrotic effects, were abundant in hCLiP-sEVs (Supplementary Figure 3A and B). We transfected TGF-β-activated HSCs with a miR-122-5p or miR-29a-3p mimic. Expression of *ACTA2* and *COL1A1* in HSCs

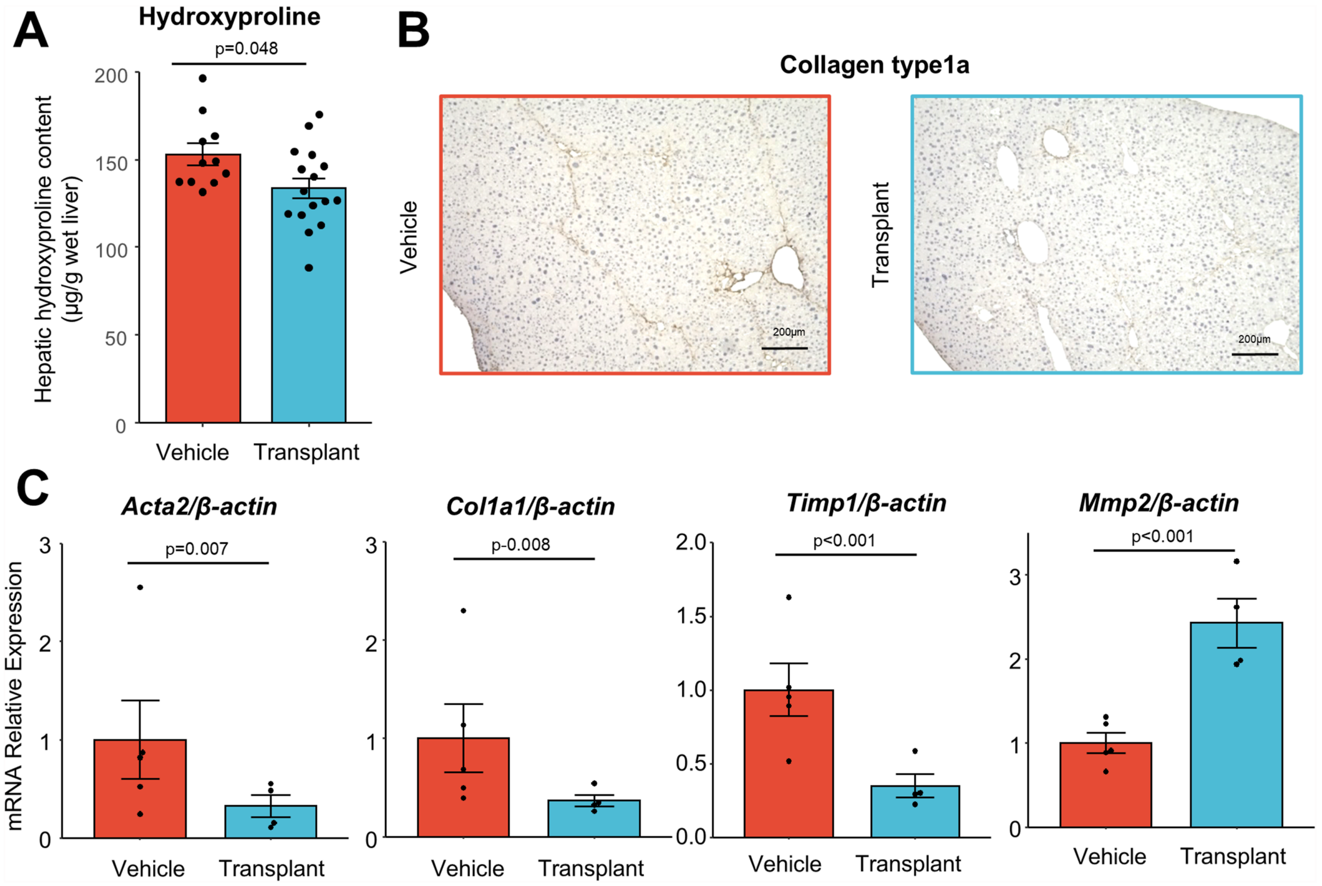

Figure 1. hCLiP transplantation improves liver fibrosis in mice. (A) The hydroxyproline content of the mouse liver was decreased in the transplantation group. (B) Immunostaining showed that transplantation reduced collagen type 1A expression. (C) mRNA expression analysis showed that transplantation upregulated *Mmp2*, an antifibrotic gene, while downregulating profibrotic genes, *Acta2*, *Col1a1*, and *Timp1*.

significantly decreased upon transfection of the miR-122-5p and miR-29a-3p mimics, respectively (Figure 2C, Supplementary Figure 3C). Finally, to determine whether miR-122-5p and miR-29a-3p in hCLiP-sEVs are responsible for the effects of hCLiPs, we transfected TGF-β-activated HSCs with a miR-122-5p or miR-29a-3p inhibitor and exposed them to hCLiP-sEVs. The decreases in expression of *ACTA2* and *COL1A1* in HSCs following hCLiP-sEV exposure were abrogated upon transfection of these miRNA inhibitors, respectively (Figure 2D).

In summary, our results indicate that hCLiPs and hCLiP-sEVs exert an antifibrotic effect through their RNA cargo, particularly miR-122-5p and miR-29a-3p (Supplementary Figure 3D). Cell-free sEV therapy may offer a safer alternative to cell transplantation, with reduced risks of immune rejection and tumorigenicity, although long-term safety profiles remain to be fully established. Therefore, sEVs have been proposed as a novel cell-free therapy for liver fibrosis, and hCLiPs are a promising antifibrotic modality. Both miR-122-5p and miR-29a-3p elicit antifibrotic effects. Furthermore, miR-122-5p is specifically expressed in the liver and is therefore absent in sEVs derived from MSCs.[10] hCLiP-sEVs are immortalized and can be stably cultured on a large scale in culture medium supplemented with small-molecule compounds, eliminating the need for specialized culture procedures. For these reasons, we propose that hCLiPs provide a more promising sEV source for clinical applications than other cell types. In this report, we revealed that exposure to hCLiP-sEVs suppressed expression of both *ACTA2* and *COL1A1*, which indicates the importance of including a diverse range of miRNAs; however, our focus was limited to 2 well-known miRNAs. Other miRNAs and mRNAs may also contribute to the antifibrotic effect of hCLiPs. While the CCl_4 model effectively mimics fibrogenesis, we acknowledge that it does not fully recapitulate the metabolic complexity of human metabolic dysfunction–associated steatotic liver disease/metabolic dysfunction–associated steatohepatitis. In the future, it will be essential to further

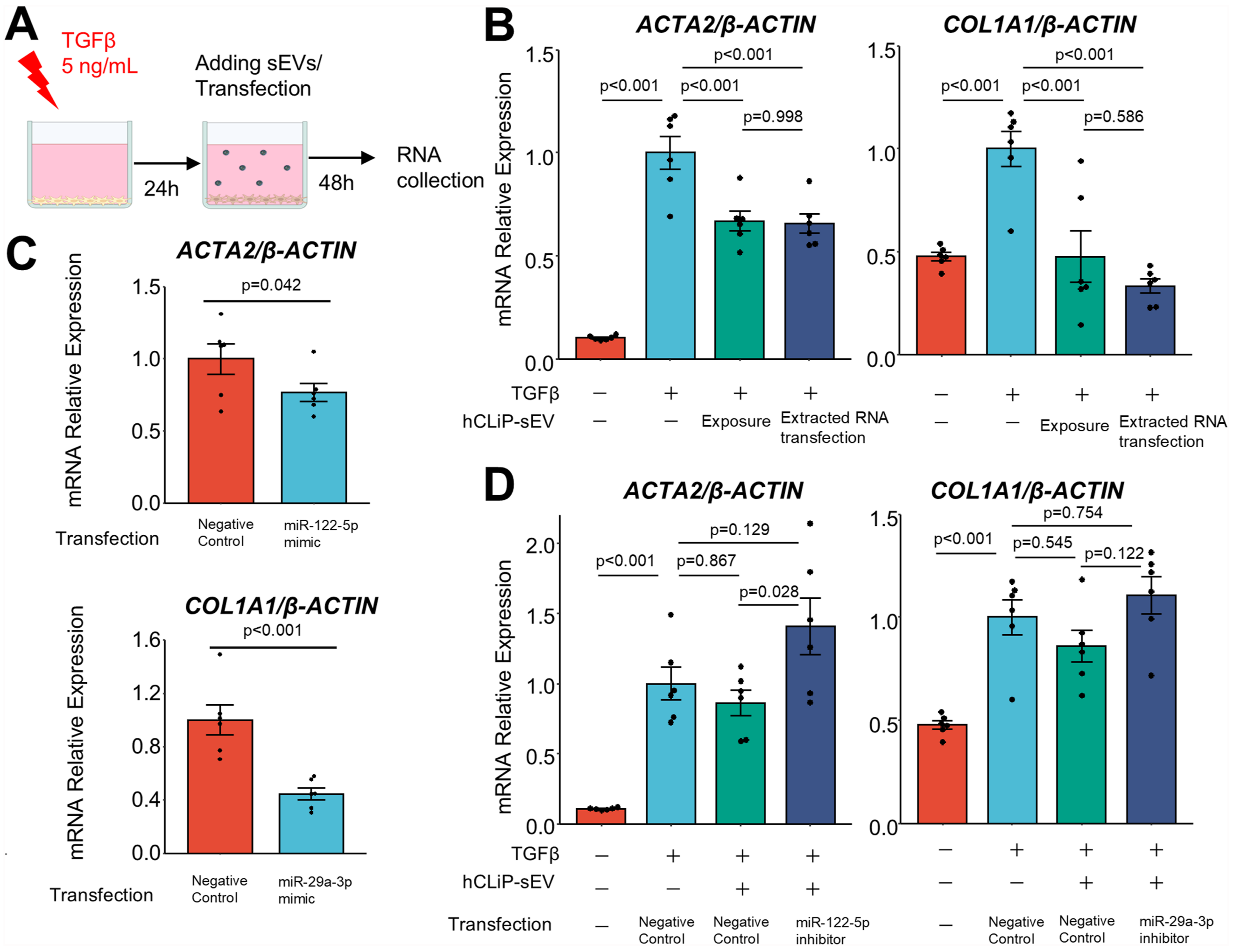

Figure 2. hCLiP-sEVs ameliorate liver fibrosis through their RNA cargo, particularly miR-122-5p and miR-29a-3p. (A) sEVs derived from immortalized hCLiPs suppressed the profibrotic phenotype of human HSCs. Transfection of total RNA extracted from hCLiP-sEVs induced similar levels of *ACTA2* and *COL1A1* expression as hCLiP-sEV exposure. (B) Schematic protocol for hCLiP-sEV exposure and transfection. (C) Transfection of the miR-122-5p and miR-29a-3p mimics to HSCs decreased the expression of *ACTA2* and *COL1A1*, respectively. (D) Transfection of a miR-122-5p and miR-29a-3p inhibitor abrogated the decrease in expression of *ACTA2* and *COL1A1* in HSCs following exposure to hCLiP-sEVs, respectively.

elucidate the mechanism of action by directly comparing miRNA therapies and hCLiP-sEVs and to determine which has the greater therapeutic effect using metabolic fibrosis models.

TOMOKO YAMAGUCHI[1]
JUNTARO MATSUZAKI[1,2,3]
TAKESHI KATSUDA[4]
KIMINORI KIMURA[5]
THE HCLIP-EV PROJECT GROUP
YOSHIMASA SAITO[1]
TAKAHIRO OCHIYA[3]

[1]Division of Pharmacotherapeutics, Keio University Faculty of Pharmacy, Tokyo, Japan
[2]Division of Interdisciplinary Genetics and Nanomedicine, Research Center for Drug Discovery, Keio University Faculty of Pharmacy, Tokyo, Japan
[3]Department of Molecular and Cellular Medicine, Tokyo Medical University, Tokyo, Japan
[4]Department of Chemical System Engineering, Graduate School of Engineering, The University of Tokyo, Tokyo, Japan
[5]Department of Hepatology, Tokyo Metropolitan Cancer and Infectious Diseases Center, Komagome Hospital, Tokyo, Japan

Correspondence:
Address correspondence to: Juntaro Matsuzaki, MD, PhD, Division of Interdisciplinary Genetics and Nanomedicine, Research Center for Drug Discovery, Keio University Faculty of Pharmacy, 1-5-30 Shibakoen, Minato-ku, Tokyo 105-8512, Japan. e-mail: juntaro.matsuzaki@keio.jp; or Takahiro Ochiya, PhD, Department of Molecular and Cellular Medicine, Institute of Medical Science, Tokyo Medical University, 6-7-1 Nishi-Shinjuku, Shinjuku-ku, Tokyo 160-0023, Japan. e-mail: tochiya@tokyo-med.ac.jp.

Supplementary Materials

Material associated with this article can be found in the online version at https://doi.org/10.1016/j.gastha.2026.100910.

References

1. Kisseleva T, et al. Nat Rev Gastroenterol Hepatol 2021;18(3):151–166.
2. Katsuda T, et al. Cell Stem Cell 2017;20(1):41–55.
3. Murakami S, et al. Regen Ther 2022;21:574–583.

4. Miyamoto D, et al. J Hepatobiliary Pancreat Sci 2024;31(10):697–704.
5. Katsuda T, et al. Elife 2019;8: e47313.
6. Sitbon A, et al. J Transl Med 2024; 22(1):480.
7. Takeuchi S, et al. NPJ Regen Med 2021;6(1):19.
8. Kadota T, et al. J Extracell Vesicles 2021;10(10):e12124.
9. Prieto-Vila M, et al. J Extracell Vesicles 2024;13(7):e12461.
10. Liu CH, et al. J Hepatol 2018; 69(6):1335–1348.

Abbreviations used in this paper: **CLiPs, chemically induced liver progenitors; hCLiP-sEVs, human chemically induced liver progenitor-derived small extracellular vesicles; hCLiPs, human chemically induced liver progenitors; HSCs, hepatic stellate cells; miRNA, microRNA; MSCs, mesenchymal stem cells; PCR, polymerase chain reaction; sEVs, small extracellular vesicles; TGF-β, transforming growth factor β**

Most current article

Copyright © 2026 The Authors. Published by Elsevier Inc. on behalf of the AGA Institute. This is an open access article under the CC BY license (http://creativecommons.org/licenses/by/4.0/).
2772-5723
https://doi.org/10.1016/j.gastha.2026.100910

Received October 28, 2025. Accepted February 25, 2026.

Acknowledgments:
We thank Ms Ayako Inoue and Mr Takumi Sonoda for supporting the in vivo experiments, Dr Chunyan Hua and Dr Hayato Kurata for providing technical support for digital PCR, and Dr Yusuke Yamamoto and Dr Yusuke Yoshioka for offering valuable advice.
The hCLiP-EV Project Group includes Hitoshi Tsugawa (Transkingdom Signaling Research Unit, Division of Host Defense Mechanism, Tokai University School of Medicine, Kanagawa, Japan), Tohru Kiyono (Project for Prevention of HPV-Related Cancer, Exploratory Oncology Research and Clinical Trial Center, National Cancer Center, Chiba, Japan), Masaki Kimura (Division of Pharmacotherapeutics, Keio University Faculty of Pharmacy, Tokyo, Japan), Marta Prieto-Vila (Division of Interdisciplinary Genetics and Nanomedicine, Research Center for Drug Discovery, Keio University Faculty of Pharmacy, Tokyo, Japan and Department of Molecular and Cellular Medicine, Tokyo Medical University, Tokyo, Japan), Yuto Oshima (Division of Pharmacotherapeutics, Keio University Faculty of Pharmacy, Tokyo, Japan), and Yoshitaka Kawamoto (Division of Pharmacotherapeutics, Keio University Faculty of Pharmacy, Tokyo, Japan).

Authors' Contributions:
Tomoko Yamaguchi: Conceptualization: equal; data curation: lead; formal analysis: lead; investigation: lead; methodology: lead; visualization: lead; writing – original draft: lead; writing – review and editing: equal. Juntaro Matsuzaki: Conceptualization: equal; data curation: equal; funding acquisition: equal; methodology: equal; writing – original draft: equal; writing – review and editing: lead. Takeshi Katsuda: Methodology: equal; writing – review and editing: equal. Kiminori Kimura: Methodology: equal; writing – review and editing: equal. Yoshimasa Saito: Methodology: equal; writing – review and editing: equal. Takahiro Ochiya: Conceptualization: lead; funding acquisition: lead; methodology: equal; writing – review and editing: equal.

Conflicts of Interest:
These authors disclose the following: Juntaro Matsuzaki, Kiminori Kimura, and Takahiro Ochiya have collaborated with Evia Life Sciences Inc. The company had no role in the study design, data collection and analysis, publication decision, or manuscript preparation. The remaining authors disclose no conflicts.

Funding:
This work was supported by Grants-in-Aid from the Research Program on Hepatitis of the Japan Agency for Medical Research and Development (16fk0310512h0005 [to Takahiro Ochiya], 17fk0310101h0001 [to Takahiro Ochiya], 24fk0210148h0001 [to Juntaro Matsuzaki], 25fk0210148h0002 [to Juntaro Matsuzaki]), the Promotion and Mutual Aid Corporation for Private Schools of Japan (to Juntaro Matsuzaki), and a grant from Astellas Pharma Inc (to Takahiro Ochiya) and Evia Life Sciences Inc (to Takahiro Ochiya and Juntaro Matsuzaki).

Ethical Statement:
Animal experimental procedures were approved by and performed in accordance with the Keio University Institutional Animal Care and Use Committee (license number, A2021–053).

Data Transparency Statement:
Data will be supplied on reasonable request to the corresponding author.

Reporting Guidelines:
All animal-related experiments adhere to standards articulated in the Animal Research: Reporting of In Vivo Experiments (ARRIVE) and the Institutional Guidelines on Animal Experimentation at Keio University.

Gastro Hep Advances, Volume 5

Supplemental information

Small Extracellular Vesicles Released From Human Chemically Induced Liver Progenitors Have the Potential to Improve Liver Fibrosis in Mice

Tomoko Yamaguchi, Juntaro Matsuzaki, Takeshi Katsuda, Kiminori Kimura, The hCLiP-EV Project GroupYoshimasa Saito, and Takahiro Ochiya

Supplementary Materials

Cell culture

Primary human hepatocytes (PHHs) (lot FCL) were purchased from Veritas Corporation. The basal medium for culture of PHHs (SHM medium) was prepared following a previous report[1]. Depending on the experiment, this basal medium for hCLiPs was supplemented with 10% KnockOut Serum Replacement (Thermo Scientific) and small molecules, namely, 0.5 μM A-83-01 (Wako) and 3 μM CHIR99021 (Selleck). HHSteC were purchased from ScienCell Research Laboratories and maintained in Stellate Cell Medium containing Stellate Cell Growth Supplement, 2% fetal bovine serum, and penicillin/streptomycin (P/S) (Science Cell Research Laboratories). LX-2 cells were purchased from Sigma and maintained in DMEM, high glucose (Nacalai Tesque, Inc.) containing fetal bovine serum (Biowest) amd P/S (Nacalai Tesque, Inc.).

Induction of hCLiPs from PHHs

hCLiPs were generated from PHHs (lot FCL), which has high hepatic identity in a previous report[1].

hCLiP transplantation into mice with liver fibrosis

To induce liver fibrosis, CCl_4 (0.5 mL/kg) (Wako) dissolved in olive oil (Wako) was administered intraperitoneally twice per week for 8 weeks to 8-week-old NOD-SCID mice. hCLiPs were pelleted

using TrypLE Express (Life Technologies) and suspended in DMEM (Life Technologies). Under isoflurane (Pfizer) anesthesia, the spleen was exposed and 5 × 10^5 cells were injected into each mouse. Two weeks after transplantation, mice were sacrificed and the degree of liver fibrosis was evaluated. The dosage and schedule were determined based on established protocols for liver cell transplantation[2].

Hydroxyproline quantification

Hydroxyproline in liver tissue was quantified using a Hydroxyproline Assay Kit (BioVision) according to the manufacturer's instructions.

Digital PCR

Total DNA was extracted from frozen liver tissue using a DNeasy Blood & Tissue Kit (Qiagen). To analyze human cells in mouse liver after hCLiP transplantation, mouse liver following hCLiP transplantation were analyzed using total DNA, the TaqMan Copy Number Reference Assay, Mouse Tfrc (VIC), and probes from a TaqMan RNase P Detection Reagents Kit (FAM). Digital PCR detection was performed using the QuantStudio 3D Digital PCR System (Thermo Fisher Scientific) with PCR Master Mix v2.

Immunohistochemistry

Following dewaxing and rehydration, heat-induced epitope retrieval was performed in ImmunoSaver (Nissin EM) at 98°C for 45 min. Endogenous peroxidase was inactivated with methanol containing 0.3% H_2O_2 at room temperature for 30 min. Thereafter, specimens were permeabilized with 0.1% Triton X-100, treated with Blocking One solution at 4°C for 30 min, and incubated with primary antibodies at 4°C overnight (Supplementary Table). Sections were stained using ImmPRESS IgG-Peroxidase Kits (Vector Labs) and a Metal Enhanced DAB Substrate Kit (Life Technologies) according to the manufacturers' instructions.

Establishment of immortalized hCLiPs

hCLiPs were immortalized by overexpressing the $CDK4^{R24C}$, Cyclin D1, and TERT genes located downstream of the EpCAM promoter using the lentiviral vector plasmids CSII-EpCAMp-tetOff-Adv, CSII-TRE-Tight-cyclin D1, and CSII-TRE-Tight-$CDK4^{R24C}$ [3]. Cell sorting was performed using a FACSAria Ⅲ instrument (BD Biosciences). Cells were labeled with propidium iodide (BD Biosciences) and sorted into 96-well plates.

Albumin enzyme-linked immunosorbent assay

Human albumin in the culture supernatant was quantified using a Human Albumin ELISA

Quantitation Kit (Bethyl) according to the manufacturer's instructions.

Collection of sEVs

hCLiPs were suspended in SHM medium containing 10% KnockOut Serum Replacement, A-83-01, and CHIR99021, and seeded at a density of 3×10^4 viable cells/cm^2. The medium was replaced with SHM containing A-83-01 and CHIR99021 (without KnockOut Serum Replacement) on the second day of culture. The culture supernatant was collected after 48 h of culture and centrifuged at 2,000 g for 10 min at 4°C, and the supernatant was filtered using a 0.22 μm filter. The pretreated culture supernatant was ultracentrifuged at 35,000 rpm for 1 h and 10 min at 4℃. Immediately after ultracentrifugation, the supernatant was discarded and sEVs were pelleted (ultracentrifugation was sometimes repeated depending on the amount of culture supernatant). The pellet was resuspended in phosphate-buffered saline (PBS), the sample was ultracentrifuged again, and the supernatant was discarded. The pellet was washed and resuspended in the small amount of PBS left in the tube to create the sEV solution. The collection method, validation, and quantification methods suffice the MISEV2023 guidelines[4].

Nanoparticle tracking analysis

To quantify the particle number in EV samples, nanoparticle tracking analysis was performed

using NanoSight NS300.

Transmission electron microscopy (TEM)

hCLiP-sEVs were fixed by resuspending them in TEM sample buffer containing 1% glutaraldehyde followed by incubation overnight at 4°C. The sample was pipetted onto a Formvar-coated 200-mesh nickel grid (Ted Pella Inc.) and allowed to settle for 25 min. The grid was air-dried and then TEM images were obtained using a JEM-1400 transmission electron microscope (JEOL) at 100 kV.

Protein extraction

Cells were lysed with M-PER Mammalian Protein Extraction Reagent (Thermo Fisher Scientific) by thoroughly pipetting. The lysate was centrifuged at 15,000 g for 10 min at 4°C, and the supernatant was used as the protein solution. The protein concentration was measured using a Pierce BCA Protein Assay Kit (Thermo Fisher Scientific) according to the manufacturer's instructions.

Immunoblotting

The protein solution was mixed with 4× SDS Sample Buffer (Millipore) and incubated at 95°C for

5 min. The sample and molecular weight marker were loaded into 4–20% Mini-PROTEAN TGX Precast Protein Gels (Bio-Rad). Electrophoresis was performed for 45 min at 150 V. Gels were transferred to an Immobilon-P membrane (Merck) for 1 h at 100 V. Blocking was performed and the membrane was incubated with the diluted primary antibody overnight at 4°C (Supplementary Table). After washing, the membrane was incubated with the diluted secondary antibody for 1 h at room temperature. The membrane was washed and stained with ECL Select Western Blotting Detection Reagent (Cytiva). Signals were detected with a Molecular Imager ChemiDoc XRS System (Bio-Rad).

miRNA-sequencing

Libraries were constructed using a QIAseq miRNA Library Kit (Qiagen) according to the manufacturer’s protocols. The pooled libraries were sequenced using QIAseq miRNA NGS 96 Index IL (Qiagen) in 75-bp single-end reads. Then, the original FASTQ files generated by CLC Genomics Workbench 22.0.2 were aligned to the miRBase v22 databank.

Exposure of HSCs to hCLiP-sEVs

HHSteCs were cultured overnight and then the medium was replaced with Stellate Cell Medium containing P/S and TGF-β (5 ng/mL) (PeproTech Inc.). After incubation for 24 h, HHSteCs were

exposed to hCLiP-sEVs for 48 h.

Transfection

Transfection of hsa-miR-122-5p inhibitor (MH11012, Thermo Fisher Scientific), hsa-miR-29a-3p inhibitor (MH12499, Thermo Fisher Scientific), hsa-miR-122-5p mimic (MC11012, Thermo Fisher Scientific), and hsa-miR-29a-3p mimic (MC12499, Thermo Fisher Scientific) was performed using jetPRIME (Polyplus) according to the manufacturer’s instructions.

qRT-PCR

Total RNA was isolated using an miRNeasy Mini Kit (Qiagen). Reverse transcription was performed using a High-Capacity cDNA Reverse Transcription Kit (Life Technologies) according to the manufacturer’s instructions. cDNA was used for PCR with PowerUp SYBR Green Master Mix (Applied Biosystems) using specific primers (Mmp2 forward, ACACTTTCTATGGCTGCCCC; Mmp2 reverse, GTTTCAGGGTCCAGGTCAGG; Timp1 forward, GTAATGCGTCCAGGAAGCCT; Timp1 reverse, GGGGGCCATCATGGTATCTG; Acta2 forward GGCATCATCACCAACTGGGA; Acta2 reverse, AGAGGCATAGAGGGACAGCA; Col1a forward, TTCTCCTGGCAAAGACGGAC; Col1a reverse, CTCAAGGTCACGGTCACGAA; β-actin forward, TCGTGCGTGACATCAAAGAGA; β-actin reverse, GCCACAGGATTCCATACCCAA; β-ACTIN

forward, AGCACTGTGTTGGCGTACAG; β-ACTIN reverse, ACTCTTCCAGCCTTCCTTCC; ACTA2 forward, CTGTTCCAGCCATCCTTCAT; ACTA2 reverse, GGCAATGCCAGGGTACATAG; COL1A1 forward, AACATGACCAAAAACCAAAAGTG; and COL1A1 reverse, CATTGTTTCCTGTGTCTTCTGG).

Statistics

Two groups were compared using the T test. Three groups were compared using a one-way analysis of variance, and, assuming equality of variance was suspected, Tukey’s test was performed with the linear mixed models using IBM SPSS Statistics 23 (SPSS Inc.).

Supplementary Figure Legends

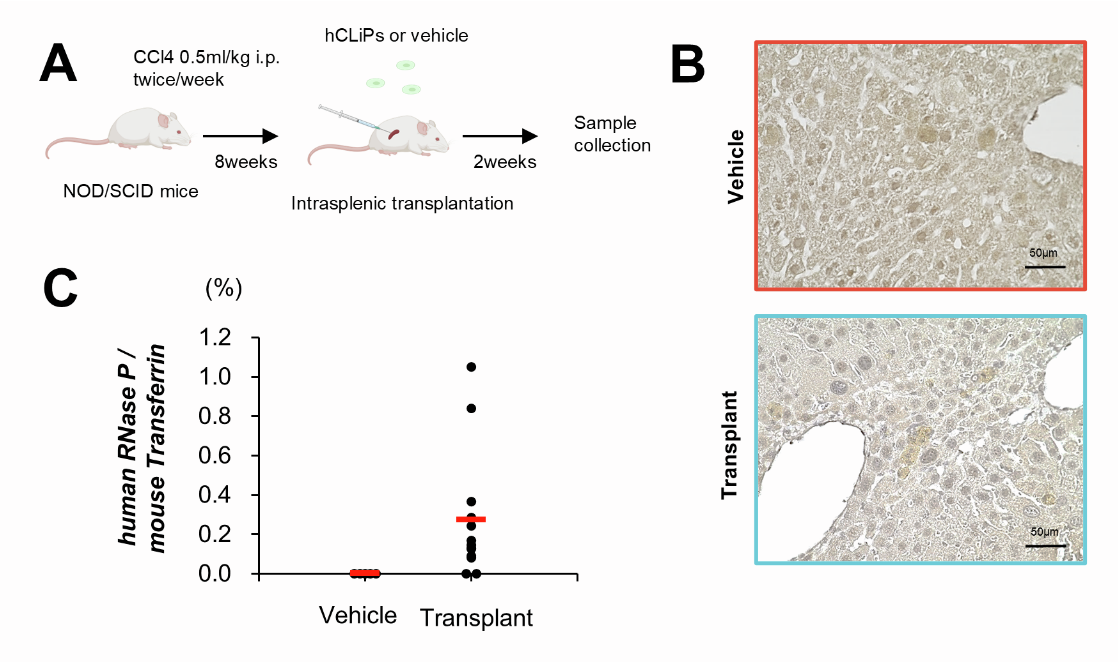

Supplementary Figure 1. The detection of human cells in the mouse tissues confirmed the successful engraftment of the transplanted hCLiP.

(A) Schematic protocol for hCLiP transplantation in a mouse model of liver fibrosis. (B) Immunohistochemistry of human mitochondria. (C) ddPCR analysis of human RNase P/mouse transferrin expression.

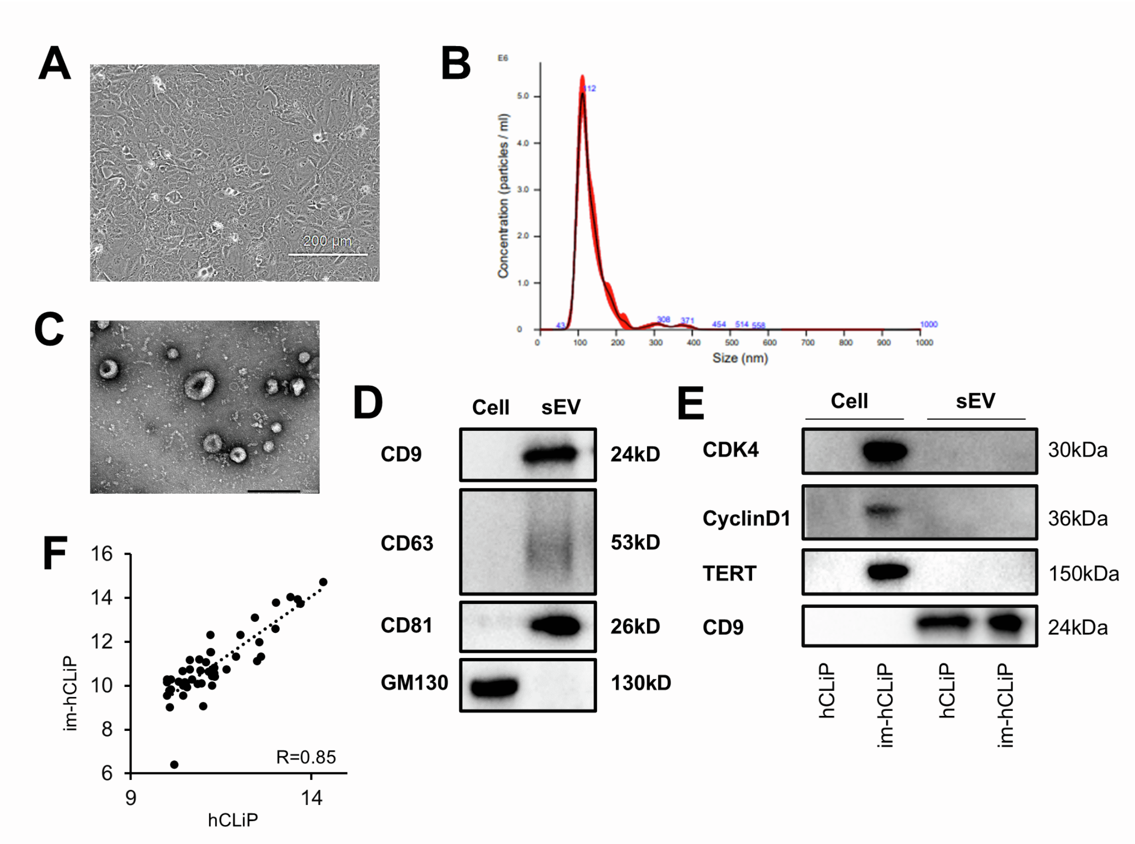

Supplementary Figure 2. Immortalized hCLiP allowed for the proper collection of sEVs, which exhibited characteristics comparable to the original hCLiP.

(A) Cell morphology. (B) Nanoparticle tracking analysis. (C) TEM. (D) Immunoblot of EV marker proteins. (E) Immunoblot of overexpressed genes. (F) miRNA sequencing.

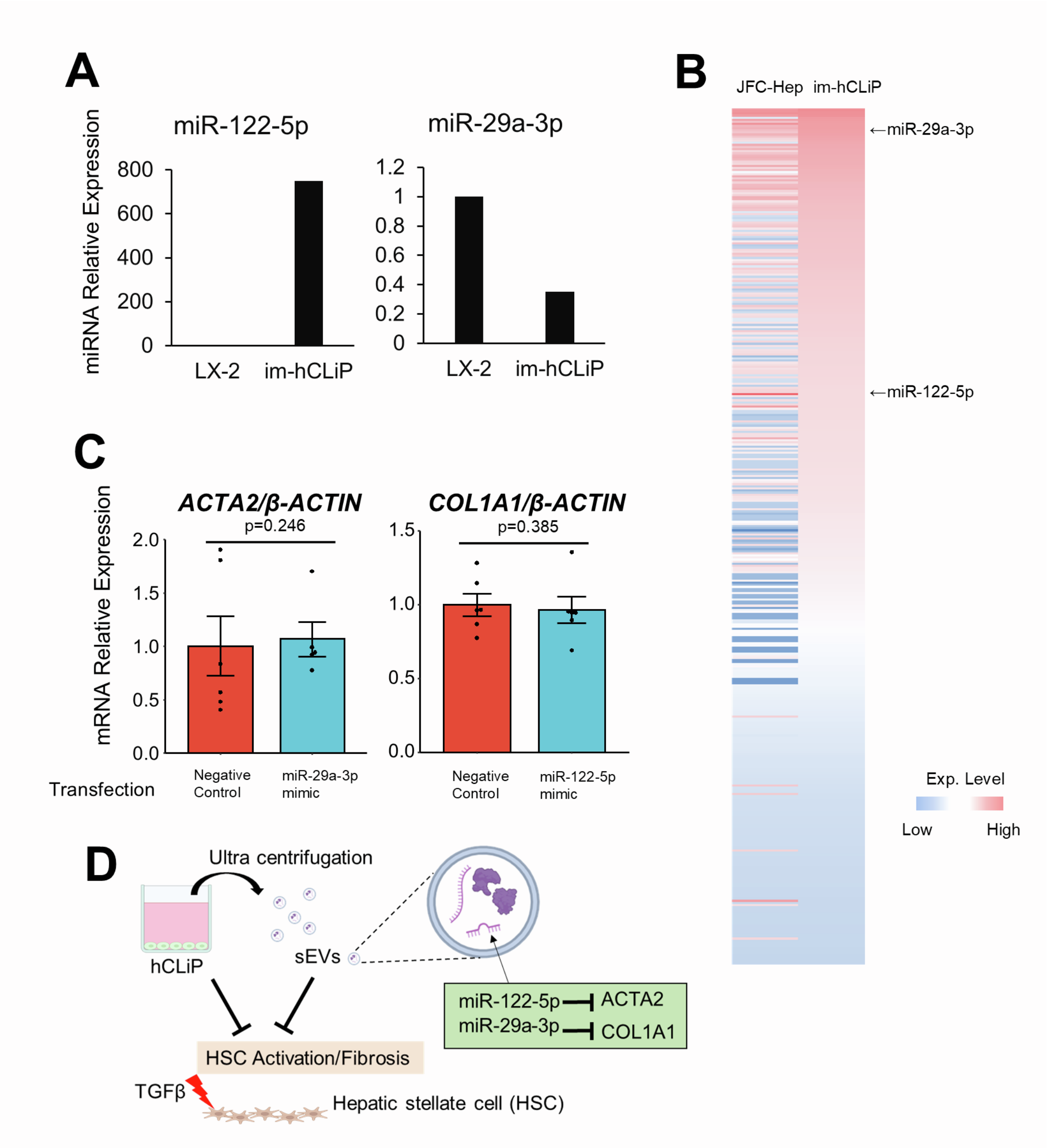

Supplementary Figure 3. The mechanism of action of hCLiP is mainly through EVs, with miR-122-5p and miR-29a-3p playing key roles in this mechanism.

(A) qPCR analysis of miR-122-5p and miR-29a-3p expression. (B) miRNA sequencing. (C) Transfection of a miR-122-5p and miR-29a-3p mimic did not affect expression of *COL1A1* and *ACTA2*, respectively. (D) Schematic overview of the study design.

Supplementary Table. List of antibodies using for immunohistochemistry and immunoblotting.

Antibody	Host animal	Catalog #	Dilution	Dilution buffer	Manufacturer
Col1a	Goat	1310-01	1/200	DAKO real diluent	Southern Biotech
CD9	Mouse	312102	1/5000	Primary: TBS-T containing 10% Blocking One solution<br>Secondary: TBS-T	BD Biosciences
CD63	Mouse	556019	1/2000	Primary: TBS-T containing 10% Blocking One solution<br>Secondary: TBS-T	BD Biosciences
CD81	Mouse	555675	1/5000	Primary: TBS-T containing 10% Blocking One solution<br>Secondary: TBS-T	BioLegend
GM130	Rabbit	11308-1-AP	1/5000	Primary: TBS-T containing 10% Blocking One solution<br>Secondary: TBS-T	Proteintech
CDK4	Rabbit	12790S	1/1000	Primary: TBS-T containing 10% Blocking One solution<br>Secondary: TBS-T	Cell Signaling
Cyclin D1	Mouse	554180	1/1000	Primary: Can Get Signal Solution 1<br>Secondary: Can Get Signal Solution 2	BD Biosciences
TERT	Rabbit	ABE2075	1/1000	Primary: 3% skim milk solution prepared in PBS-T<br>Secondary: 3% skim milk solution prepared in PBS-T	Sigma

References

1. Katsuda T, Matsuzaki J, Yamaguchi T, et al. Generation of human hepatic progenitor cells with regenerative and metabolic capacities from primary hepatocytes. Elife 2019;8:e47313, doi:10.7554/eLife.47313

2. Watanabe Y, Tsuchiya A, Seino S, et al. Mesenchymal Stem Cells and Induced Bone Marrow-Derived Macrophages Synergistically Improve Liver Fibrosis in Mice. Stem Cells Transl Med 2019;8(3):271-284, doi:10.1002/sctm.18-0105

3. Nishiwaki M, Toyoda M, Oishi Y, et al. Immortalization of human hepatocytes from biliary atresia with CDK4. Sci Rep 2020;10(1):17503, doi:10.1038/s41598-020-73992-3

4. Welsh JA, Goberdhan DCI, O'Driscoll L, et al. Minimal information for studies of extracellular vesicles (MISEV2023): From basic to advanced approaches. J Extracell Vesicles 2024;13(2):e12404, doi:10.1002/jev2.12404

Supplementary Materials

Cell culture

Primary human hepatocytes (PHHs) (lot FCL) were purchased from Veritas Corporation. The basal medium for culture of PHHs (SHM medium) was prepared following a previous report[1]. Depending on the experiment, this basal medium for hCLiPs was supplemented with 10% KnockOut Serum Replacement (Thermo Scientific) and small molecules, namely, 0.5 μM A-83-01 (Wako) and 3 μM CHIR99021 (Selleck). HHSteC were purchased from ScienCell Research Laboratories and maintained in Stellate Cell Medium containing Stellate Cell Growth Supplement, 2% fetal bovine serum, and penicillin/streptomycin (P/S) (Science Cell Research Laboratories). LX-2 cells were purchased from Sigma and maintained in DMEM, high glucose (Nacalai Tesque, Inc.) containing fetal bovine serum (Biowest) amd P/S (Nacalai Tesque, Inc.).

Induction of hCLiPs from PHHs

hCLiPs were generated from PHHs (lot FCL), which has high hepatic identity in a previous report[1].

hCLiP transplantation into mice with liver fibrosis

To induce liver fibrosis, CCl_4 (0.5 mL/kg) (Wako) dissolved in olive oil (Wako) was administered intraperitoneally twice per week for 8 weeks to 8-week-old NOD-SCID mice. hCLiPs were pelleted

using TrypLE Express (Life Technologies) and suspended in DMEM (Life Technologies). Under isoflurane (Pfizer) anesthesia, the spleen was exposed and 5 × 10^5 cells were injected into each mouse. Two weeks after transplantation, mice were sacrificed and the degree of liver fibrosis was evaluated. The dosage and schedule were determined based on established protocols for liver cell transplantation[2].

Hydroxyproline quantification

Hydroxyproline in liver tissue was quantified using a Hydroxyproline Assay Kit (BioVision) according to the manufacturer's instructions.

Digital PCR

Total DNA was extracted from frozen liver tissue using a DNeasy Blood & Tissue Kit (Qiagen). To analyze human cells in mouse liver after hCLiP transplantation, mouse liver following hCLiP transplantation were analyzed using total DNA, the TaqMan Copy Number Reference Assay, Mouse Tfrc (VIC), and probes from a TaqMan RNase P Detection Reagents Kit (FAM). Digital PCR detection was performed using the QuantStudio 3D Digital PCR System (Thermo Fisher Scientific) with PCR Master Mix v2.

Immunohistochemistry

Following dewaxing and rehydration, heat-induced epitope retrieval was performed in ImmunoSaver (Nissin EM) at 98°C for 45 min. Endogenous peroxidase was inactivated with methanol containing 0.3% H_2O_2 at room temperature for 30 min. Thereafter, specimens were permeabilized with 0.1% Triton X-100, treated with Blocking One solution at 4°C for 30 min, and incubated with primary antibodies at 4°C overnight (Supplementary Table). Sections were stained using ImmPRESS IgG-Peroxidase Kits (Vector Labs) and a Metal Enhanced DAB Substrate Kit (Life Technologies) according to the manufacturers' instructions.

Establishment of immortalized hCLiPs

hCLiPs were immortalized by overexpressing the CDK4^{R24C}, Cyclin D1, and TERT genes located downstream of the EpCAM promoter using the lentiviral vector plasmids CSII-EpCAMp-tetOff-Adv, CSII-TRE-Tight-cyclin D1, and CSII-TRE-Tight-CDK4^{R24C} [3]. Cell sorting was performed using a FACSAria Ⅲ instrument (BD Biosciences). Cells were labeled with propidium iodide (BD Biosciences) and sorted into 96-well plates.

Albumin enzyme-linked immunosorbent assay

Human albumin in the culture supernatant was quantified using a Human Albumin ELISA

Quantitation Kit (Bethyl) according to the manufacturer's instructions.

Collection of sEVs

hCLiPs were suspended in SHM medium containing 10% KnockOut Serum Replacement, A-83-01, and CHIR99021, and seeded at a density of 3 × 10^4 viable cells/cm^2. The medium was replaced with SHM containing A-83-01 and CHIR99021 (without KnockOut Serum Replacement) on the second day of culture. The culture supernatant was collected after 48 h of culture and centrifuged at 2,000 g for 10 min at 4°C, and the supernatant was filtered using a 0.22 μm filter. The pretreated culture supernatant was ultracentrifuged at 35,000 rpm for 1 h and 10 min at 4℃. Immediately after ultracentrifugation, the supernatant was discarded and sEVs were pelleted (ultracentrifugation was sometimes repeated depending on the amount of culture supernatant). The pellet was resuspended in phosphate-buffered saline (PBS), the sample was ultracentrifuged again, and the supernatant was discarded. The pellet was washed and resuspended in the small amount of PBS left in the tube to create the sEV solution. The collection method, validation, and quantification methods suffice the MISEV2023 guidelines[4].

Nanoparticle tracking analysis

To quantify the particle number in EV samples, nanoparticle tracking analysis was performed

using NanoSight NS300.

Transmission electron microscopy (TEM)

hCLiP-sEVs were fixed by resuspending them in TEM sample buffer containing 1% glutaraldehyde followed by incubation overnight at 4°C. The sample was pipetted onto a Formvar-coated 200-mesh nickel grid (Ted Pella Inc.) and allowed to settle for 25 min. The grid was air-dried and then TEM images were obtained using a JEM-1400 transmission electron microscope (JEOL) at 100 kV.

Protein extraction

Cells were lysed with M-PER Mammalian Protein Extraction Reagent (Thermo Fisher Scientific) by thoroughly pipetting. The lysate was centrifuged at 15,000 g for 10 min at 4°C, and the supernatant was used as the protein solution. The protein concentration was measured using a Pierce BCA Protein Assay Kit (Thermo Fisher Scientific) according to the manufacturer's instructions.

Immunoblotting

The protein solution was mixed with 4× SDS Sample Buffer (Millipore) and incubated at 95°C for

5 min. The sample and molecular weight marker were loaded into 4–20% Mini-PROTEAN TGX Precast Protein Gels (Bio-Rad). Electrophoresis was performed for 45 min at 150 V. Gels were transferred to an Immobilon-P membrane (Merck) for 1 h at 100 V. Blocking was performed and the membrane was incubated with the diluted primary antibody overnight at 4°C (Supplementary Table). After washing, the membrane was incubated with the diluted secondary antibody for 1 h at room temperature. The membrane was washed and stained with ECL Select Western Blotting Detection Reagent (Cytiva). Signals were detected with a Molecular Imager ChemiDoc XRS System (Bio-Rad).

miRNA-sequencing

Libraries were constructed using a QIAseq miRNA Library Kit (Qiagen) according to the manufacturer's protocols. The pooled libraries were sequenced using QIAseq miRNA NGS 96 Index IL (Qiagen) in 75-bp single-end reads. Then, the original FASTQ files generated by CLC Genomics Workbench 22.0.2 were aligned to the miRBase v22 databank.

Exposure of HSCs to hCLiP-sEVs

HHSteCs were cultured overnight and then the medium was replaced with Stellate Cell Medium containing P/S and TGF-β (5 ng/mL) (PeproTech Inc.). After incubation for 24 h, HHSteCs were

exposed to hCLiP-sEVs for 48 h.

Transfection

Transfection of hsa-miR-122-5p inhibitor (MH11012, Thermo Fisher Scientific), hsa-miR-29a-3p inhibitor (MH12499, Thermo Fisher Scientific), hsa-miR-122-5p mimic (MC11012, Thermo Fisher Scientific), and hsa-miR-29a-3p mimic (MC12499, Thermo Fisher Scientific) was performed using jetPRIME (Polyplus) according to the manufacturer's instructions.

qRT-PCR

Total RNA was isolated using an miRNeasy Mini Kit (Qiagen). Reverse transcription was performed using a High-Capacity cDNA Reverse Transcription Kit (Life Technologies) according to the manufacturer's instructions. cDNA was used for PCR with PowerUp SYBR Green Master Mix (Applied Biosystems) using specific primers (Mmp2 forward, ACACTTTCTATGGCTGCCCC; Mmp2 reverse, GTTTCAGGGTCCAGGTCAGG; Timp1 forward, GTAATGCGTCCAGGAAGCCT; Timp1 reverse, GGGGGCCATCATGGTATCTG; Acta2 forward GGCATCATCACCAACTGGGA; Acta2 reverse, AGAGGCATAGAGGGACAGCA; Col1a forward, TTCTCCTGGCAAAGACGGAC; Col1a reverse, CTCAAGGTCACGGTCACGAA; β-actin forward, TCGTGCGTGACATCAAAGAGA; β-actin reverse, GCCACAGGATTCCATACCCAA; β-ACTIN

forward, AGCACTGTGTTGGCGTACAG; β-ACTIN reverse, ACTCTTCCAGCCTTCCTTCC; ACTA2 forward, CTGTTCCAGCCATCCTTCAT; ACTA2 reverse, GGCAATGCCAGGGTACATAG; COL1A1 forward, AACATGACCAAAAACCAAAAGTG; and COL1A1 reverse, CATTGTTTCCTGTGTCTTCTGG).

Statistics

Two groups were compared using the T test. Three groups were compared using a one-way analysis of variance, and, assuming equality of variance was suspected, Tukey’s test was performed with the linear mixed models using IBM SPSS Statistics 23 (SPSS Inc.).

Supplementary Figure Legends

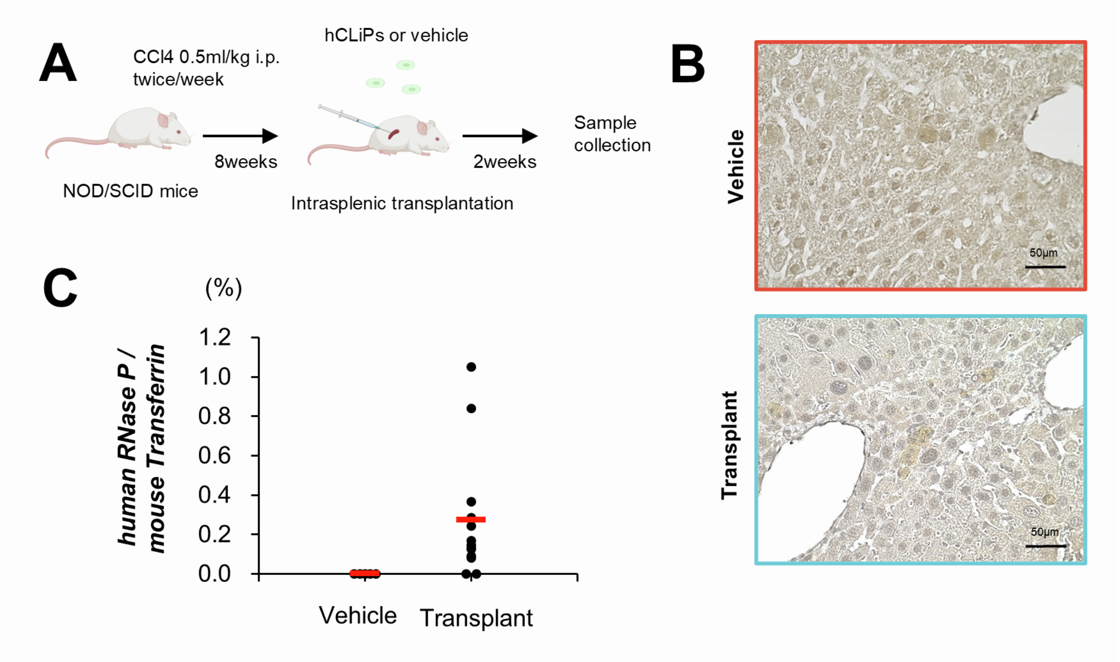

Supplementary Figure 1. The detection of human cells in the mouse tissues confirmed the successful engraftment of the transplanted hCLiP.

(A) Schematic protocol for hCLiP transplantation in a mouse model of liver fibrosis. (B) Immunohistochemistry of human mitochondria. (C) ddPCR analysis of human RNase P/mouse transferrin expression.

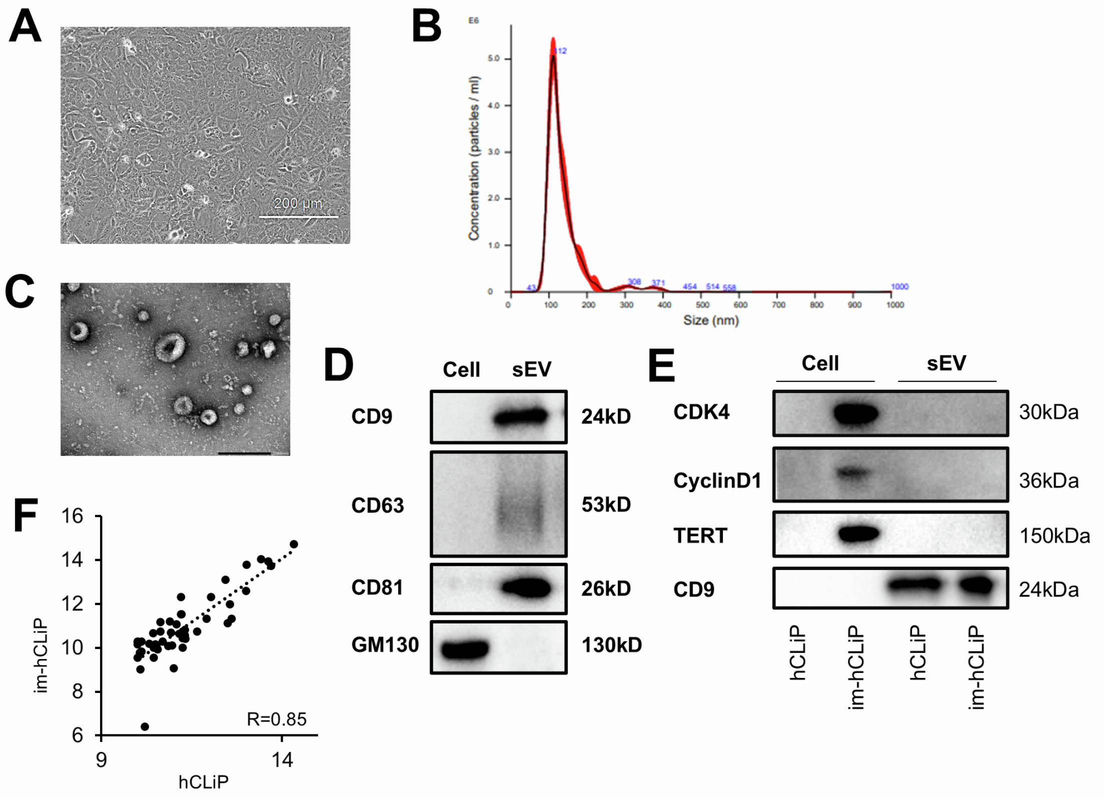

Supplementary Figure 2. Immortalized hCLiP allowed for the proper collection of sEVs, which exhibited characteristics comparable to the original hCLiP.

(A) Cell morphology. (B) Nanoparticle tracking analysis. (C) TEM. (D) Immunoblot of EV marker proteins. (E) Immunoblot of overexpressed genes. (F) miRNA sequencing.

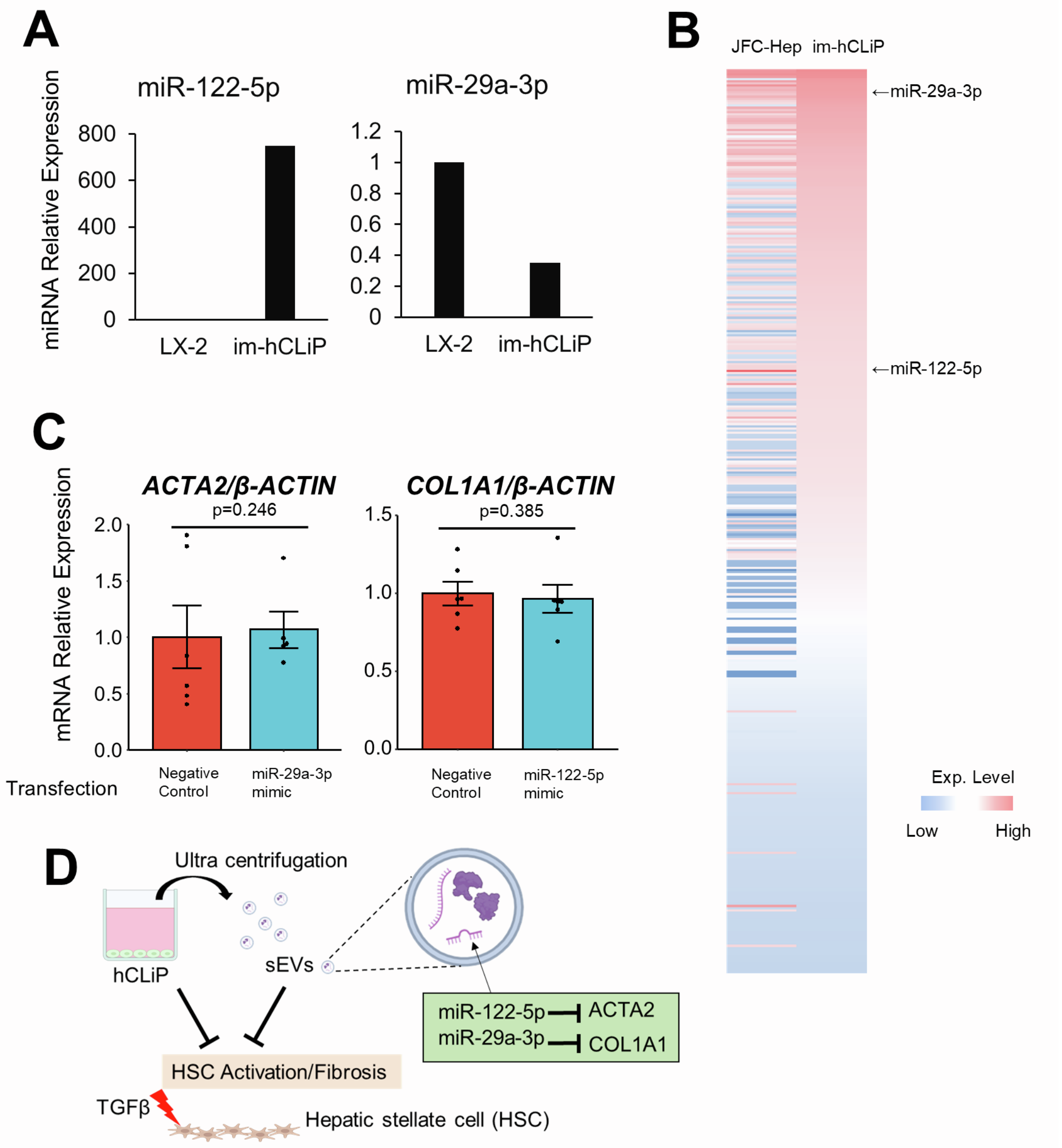

Supplementary Figure 3. The mechanism of action of hCLiP is mainly through EVs, with miR-122-5p and miR-29a-3p playing key roles in this mechanism.

(A) qPCR analysis of miR-122-5p and miR-29a-3p expression. (B) miRNA sequencing. (C) Transfection of a miR-122-5p and miR-29a-3p mimic did not affect expression of *COL1A1* and *ACTA2*, respectively. (D) Schematic overview of the study design.

Supplementary Table. List of antibodies using for immunohistochemistry and immunoblotting.

Antibody	Host animal	Catalog #	Dilution	Dilution buffer	Manufacturer
Col1a	Goat	1310-01	1/200	DAKO real diluent	Southern Biotech
CD9	Mouse	312102	1/5000	Primary: TBS-T containing 10% Blocking One solution<br>Secondary: TBS-T	BD Biosciences
CD63	Mouse	556019	1/2000	Primary: TBS-T containing 10% Blocking One solution<br>Secondary: TBS-T	BD Biosciences
CD81	Mouse	555675	1/5000	Primary: TBS-T containing 10% Blocking One solution<br>Secondary: TBS-T	BioLegend
GM130	Rabbit	11308-1-AP	1/5000	Primary: TBS-T containing 10% Blocking One solution<br>Secondary: TBS-T	Proteintech
CDK4	Rabbit	12790S	1/1000	Primary: TBS-T containing 10% Blocking One solution<br>Secondary: TBS-T	Cell Signaling
Cyclin D1	Mouse	554180	1/1000	Primary: Can Get Signal Solution 1<br>Secondary: Can Get Signal Solution 2	BD Biosciences
TERT	Rabbit	ABE2075	1/1000	Primary: 3% skim milk solution prepared in PBS-T<br>Secondary: 3% skim milk solution prepared in PBS-T	Sigma

References

1. Katsuda T, Matsuzaki J, Yamaguchi T, et al. Generation of human hepatic progenitor cells with regenerative and metabolic capacities from primary hepatocytes. Elife 2019;8:e47313, doi:10.7554/eLife.47313

2. Watanabe Y, Tsuchiya A, Seino S, et al. Mesenchymal Stem Cells and Induced Bone Marrow-Derived Macrophages Synergistically Improve Liver Fibrosis in Mice. Stem Cells Transl Med 2019;8(3):271-284, doi:10.1002/sctm.18-0105

3. Nishiwaki M, Toyoda M, Oishi Y, et al. Immortalization of human hepatocytes from biliary atresia with CDK4. Sci Rep 2020;10(1):17503, doi:10.1038/s41598-020-73992-3

4. Welsh JA, Goberdhan DCI, O'Driscoll L, et al. Minimal information for studies of extracellular vesicles (MISEV2023): From basic to advanced approaches. J Extracell Vesicles 2024;13(2):e12404, doi:10.1002/jev2.12404
